# Correction: Hyporheic hydraulic geometry: Conceptualizing relationships among hyporheic exchange, storage, and water age

**DOI:** 10.1371/journal.pone.0308936

**Published:** 2024-08-12

**Authors:** Geoffrey C. Poole, S. Kathleen Fogg, Scott J. O’Daniel, Byron E. Amerson, Ann Marie Reinhold, Samuel P. Carlson, Elizabeth J. Mohr, Hayley C. Oakland

The S1 Appendix is uploaded incorrectly. Please view the correct S1 Appendix below.

## Supporting information

S1 Appendix (PDF)

## References

[1] Poole GC, Fogg SK, O’Daniel SJ, Amerson BE, Reinhold AM, Carlson SP, et al. (2022) Hyporheic hydraulic geometry: Conceptualizing relationships among hyporheic exchange, storage, and water age. PLOS ONE 17(1): e0262080. 10.1371/journal.pone.0262080 35030186 PMC8759689

