# Supplementary material for: Hyporheic hydraulic geometry: Conceptualizing relationships among hyporheic exchange, storage, and water age
Source: PLoS One. 2022 Jan 14;17(1):e0262080. doi: 10.1371/journal.pone.0262080 (PMC8759689; doi:10.1371/journal.pone.0262080)
Supplement: Attachment — Submitted filename: poole_review.pdf [file pone.0262080.s002.pdf]

Review of PONE-D-21-04355: "Hyporheic hydraulic geometry"

April 16, 2021

In this study, Poole et al. use chemical engineering reactor theory to quantify residence time distribution (RTD) and the distribution of water ages. Their specific aims for this work are to introduce the theory to fluvial ecologists and resource managers, as well as highlight the relation between various metrics that describe the hyporheic zone and surface water-groundwater interactions. For the latter aim, the authors present various figures that demonstrate the relation between RTD, water age in the hyporheic zone, hyporheic exchange fluxes, and hyporheic zone storage volume for a simple alluvial aquifer parameterized with a power-law RTD. They discuss the assumptions underlying their modeling exercise.

There is no new mathematical theory introduced in the study, as its principal purpose is to broaden the application of canonical reactor theory. By discussing the relation between the various hyporheic zone river properties, the authors largely achieve this purpose, though I believe there are several concepts, clarfications, and theoretical advances (beyond Danckwerts) that would serve the reader if they were included in the discussion. I raise these points in the major comments section, as well as provide more specific comments below.

Major Comments

C01 : *Expand discussion on how the model relates to physical mechanisms controlling hyporheic exchange.* The authors' main arguments are premised largely upon several underlying assumptions: (1) a power-law residence time distribution is an appropriate choice for coarse-grained alluvial aquifers; (2) steady state discharge; (3) validity of the model is premised on appropriate choice of the integration limits of the power law distribution, which have largely been ignored by experimentalists and modelers; and (4) engineering reactor theory has largely been ignored by aquatic researchers and water managers outside of hydrology (implicit assumption).

The authors do make a concerted effort to discuss the limitations of some of their assumptions, this but their discussion largely builds on the premise that a power-law RTD is a ubiquitous feature of any system that results from the universal presence of nested hyporheic flowpaths. This is a rather restrictive premise, which implicitly assumes that advective flows dominate transport in any hyporheic zone. While this assumption is made explicitly in the introduction and may largely hold over the scales associated with Figure 2, but it likely does not hold near the limits of integration (which comprises a large part of the discussion). Power-law scaling in subsurface flows also results from diffusive exchange between mobile (in the subsurface sense) and immobile (i.e., low permeability) regions of the aquifer (*Haggerty and Gorelick*, 1995) and competition between advective and diffusive timescales can lead to a transition between tailing regimes (*Bottacin-Busolin and Marion*, 2010).

While the purpose of this simulation study is largely expositional in its aims, I believe it would be more informative if the authors enhance their discussion on the mechanisms of water transport in subsurface aquifers. I share the authors' motivation of accurately estimating hyporheic exchange flows, subsurface RTDs, and aquifer storage in order to use these metrics within a broader framework that would improve estimates of stream bioreactivity or some other ecosystem property. For this reason I encourage the authors to help the reader understand the mechanisms that may control the parameters they use to determine physical transport. The authors devote a good amount of text to explain the relation between the RTD and the magnitude of hyporheic exchange fluxes (with fixed storage

and integration limits), but the range of power law exponents explored would encompass a range of different transport mechanisms that ultimately result in completely different physical processes governing hyporheic transport. An α of 1.9 borders on a "thin-tailed" distribution that could be approximated with an exponential RTD, which represents a well mixed system (sensu *Bencala and Walters*, 1983). For an aquifer with constant storage and subsurface properties on the scale of Figure 2, this would represent very rapid transport and (likely) turbulent mixing in a coarse grained aquifer. It is unrealistic to assume that the bounds of integration on the RTD would be the same as in a system described by an RTD with $\alpha = 1.3$.

Mechanistically, it is known that both limits of integration of the power-law RTD are likely bounded by physical processes. For example, τ_0 may tend to zero if the process is molecular diffusion molecular diffusion or transition to an exponential-like RTD due to rapid turbulent mixing in coarse grained streambeds (*Roche et al.*, 2019); these are two example of cases where τ_0 is not explicitly "bounded by the scale at which geomorphic features can be represented by the model." Similarly, τ_n is set by some structural property of the aquifer such as the depth to bedrock or the advective timescale between geomorphic units (e.g., *Herzog et al.*, 2019), which also structures the transition from power law to exponential decay of the RTD at late times.

C02 : *Unclear connection to coarse grained alluvial aquifers* The reactor theory introduced here is quite general compared to more recent theory that has been developed for fluvial fate and transport. As such, it is unclear why the authors specify coarse grained aquifers throughout the study. I would expect that transport in such systems to be more dispersive than in finer grained systems for which the concept of nested flowpaths was developed, so it would be useful for the authors to provide additional details to clarify the connection to coarse grained aquifers.

C03 : *Aquifer storage is quite often unknown and very difficult to measure.* Throughout the m.s., it is assumed that s is generally known and needed to find $q_{\downarrow}$. While I rarely work at the spatial scales discussed in this study, I have found very few (if any) studies where s is faithfully measured. Usually, the exchange flux is more easily quantified, and s is unknown (e.g., *Harman et al.*, 2016), or both are unknown. The authors should include additional justification for why s is assumed known throughout this study.

C04 : *Improve connections to existing literature* I applaud the authors' encouragement of greater integration of engineering principles into ecological theory, but they would serve the reader if they acknowledge the wealth of existing work that has already used principles from reactor theory to improve models of hyporheic exchange, reactive transport, and ecological theory. Specific to hyporheic exchange, a good amount of work aiming to identify mechanistic controls on RTDs is summarized in *Boano et al.* (2014) – including links to what the authors refer to as hyporheic geometry, to reactive transport, and to ecological theory. Connection between residence time distribution and mass removal (i.e., nutrient spiraling) is, to my knowledge, first addressed in a model by *Runkel* (2007) using the phenomenological transient storage model, though this idea has been pushed forward by many others (e.g., see works by Tonina, Cardenas, and Bolster). The authors may also be interested in the developments of rSAS theory (*Harman*, 2015) and its application to hyporheic zone storage (e.g., *Harman et al.*, 2016), since rSAS theory represents a generalization of the mathematical concepts introduced here.

Specific Comments

C05 (Abstract): The main text does not describe how the "...nuanced and realistic understanding" provided in this study differs (or improves) upon current paradigms in the fields of aquatic research or resource management. Perhaps a comparison of existing approaches would help these communities better understand how they can use this theory to improve predictions, e.g., through better designed field experiments.

C06 (L23): Hyporheic discharge is more commonly referred to as hyporheic exchange flux.

C07 (L106): Terms such as isometric and unipartite will would stand to be translated if the authors are aiming to maximize adoption by aquatic researchers and water managers.

C08 (Figure 2): It is unclear how this figure was generated. What is meant by the distribution of surface water? Are advective flows considered to be moving at constant velocity, or is a physical model being used? What hyporheic zone properties are being used?

C09 (L212-215): This explanation is undercut by the fact that the choice of limits *does* influence your model results substantially, as mentioned in the Discussion. Consider replacing "for other purposes" with a brief acknowledgment of what implications the choice of integral limits has on model outcomes, since this is the intuition you hope the reader will gain.

C10 (L216): $f(\tau)$ is confusing because this notation is already used to denote the shape of the function used to create the pdf. Consider using a different variable.

C11 (L274): Note that the empirical or mechanistic relationships mentioned here must be linear for this theory to hold, which implies first-order reactions. That may be a bit of a stretch for microbial community composition, though I am unfamiliar with microbial ecology theory.

C12 (L329): "...determining the magnitude of $q_{\downarrow}$ within this model framework."

References

Bencala, K. E., and R. A. Walters (1983), Simulation of solute transport in a mountain pool-and-riffle stream: A transient storage model, *Water Resources Research*, *19*(3), 718–724, doi: 10.1029/WR019i003p00718.

Boano, F., J. W. Harvey, A. Marion, A. I. Packman, R. Revelli, L. Ridolfi, and A. Wörman (2014), Hyporheic flow and transport processes: Mechanisms, models, and biogeochemical implications, *Reviews of Geophysics*, *52*(4), 603–679, doi:10.1002/2012RG000417.

Bottacin-Busolin, A., and A. Marion (2010), Combined role of advective pumping and mechanical dispersion on time scales of bed form–induced hyporheic exchange, *Water Resources Research*, *46*(8), doi:https://doi.org/10.1029/2009WR008892.

Haggerty, R., and S. M. Gorelick (1995), Multiple-rate mass transfer for modeling diffusion and surface reactions in media with pore-scale heterogeneity, *Water Resources Research*, *31*(10), 2383–2400, publisher: Wiley Online Library.

Harman, C. J. (2015), Time-variable transit time distributions and transport: Theory and application to storage-dependent transport of chloride in a watershed, *Water Resources Research*, *51*(1), 1–30, doi:10.1002/2014WR015707.

Harman, C. J., A. S. Ward, and A. Ball (2016), How does reach-scale stream-hyporheic transport vary with discharge? Insights from rSAS analysis of sequential tracer injections in a headwater mountain stream: TIME-VARIABLE STREAM TRANSPORT, *Water Resources Research*, *52*(9), 7130–7150, doi:10.1002/2016WR018832.

Herzog, S. P., A. S. Ward, and S. M. Wondzell (2019), Multiscale Feature-feature Interactions Control Patterns of Hyporheic Exchange in a Simulated Headwater Mountain Stream, *Water Resources Research*, *55*(12), 10,976–10,992, doi:https://doi.org/10.1029/2019WR025763.

Roche, K. R., A. Li, D. Bolster, G. J. Wagner, and A. I. Packman (2019), Effects of turbulent hyporheic mixing on reach-scale transport, *Water Resources Research*, *55*(5), 3780–3795, publisher: Wiley Online Library.

Runkel, R. L. (2007), Toward a transport-based analysis of nutrient spiraling and uptake in streams, *Limnology and Oceanography: Methods*, *5*(1), 50–62.
