# Supplementary material for: Hyporheic hydraulic geometry: Conceptualizing relationships among hyporheic exchange, storage, and water age
Source: PLoS One. 2022 Jan 14;17(1):e0262080. doi: 10.1371/journal.pone.0262080 (PMC8759689; doi:10.1371/journal.pone.0262080)
Supplement: Attachment — Submitted filename: Response_To_Reviewers.pdf [file pone.0262080.s003.pdf]

Response to Review Comments

Geoffrey C. Poole, S. Kathleen Fogg, Scott J. O'Daniel, Byron E. Amerson,
Ann Marie Reinhold, Samuel P. Carlson, Elizabeth J. Mohr, and Hayley C. Oakland

August 31, 2021

1 Associate Editor Comments

New Title

Please modify the title to ensure that it is meeting PLOS' guidelines. In particular, the title should be "specific, descriptive, concise, and comprehensible to readers outside the field" and in this case it is not informative and specific about your study's scope and methodology

We have amended the title to be in compliance with the PLOS One guidance. We prefer retaining the "hydrologic geometry" reference in the title because many reader of this special issue will be familiar with the concept of hydrologic geometry in stream channels. We aim to leverage this analogy to help underscore why hydrologically accurate conceptual models of the hyporheic zone matter for stream ecology.

Data Availability

We note that you have stated that you will provide repository information for your data at acceptance. Should your manuscript be accepted for publication, we will hold it until you provide the relevant accession numbers or DOIs necessary to access your data. If you wish to make changes to your Data Availability statement, please describe these changes in your cover letter and we will update your Data Availability statement to reflect the information you provide.
We will provide repository information for the data upon acceptance.

Address copyright issue with Figure 2

We note that Figure 2 in your submission contain map/satellite images which may be copyrighted.

We re-made figure using public domain NAIP imagery.

2 Main Comments from Reviewer 2

R2 Comment 1, part 1

Expand discussion on how the model relates to physical mechanisms controlling hyporheic exchange. The authors' main arguments are premised largely upon several underlying assumptions: (1) a power-law residence time distribution is an appropriate choice for coarse-grained alluvial

aquifers; (2) steady state discharge; (3) validity of the model is premised on appropriate choice of the integration limits of the power law distribution, which have largely been ignored by experimentalists and modelers; and (4) engineering reactor theory has largely been ignored by aquatic researchers and water managers outside of hydrology (implicit assumption).

The authors do make a concerted effort to discuss the limitations of some of their assumptions, this but their discussion largely builds on the premise that a power-law RTD is a ubiquitous feature of any system that results from the universal presence of nested hyporheic flowpaths. This is a rather restrictive premise, which implicitly assumes that advective flows dominate transport in any hyporheic zone. While this assumption is made explicitly in the introduction and may largely hold over the scales associated with Figure 2, but it likely does not hold near the limits of integration (which comprises a large part of the discussion). Power-law scaling in subsurface flows also results from diffusive exchange between mobile (in the subsurface sense) and immobile (i.e., low permeability) regions of the aquifer (Haggerty and Gorelick, 1995) and competition between advective and diffusive timescales can lead to a transition between tailing regimes (Bottacin-Busolin and Marion, 2010).

We have addressed this comment in several ways. First, based on this comment and on a number of comments received (see below), we have removed references to "expansive course-grained alluvial aquifers" (except when referring to systems we study and providing citations) and simply suggest that our analysis applies to hyporheic zones in general. This seems to be the consensus of the reviewers. Second, we have modified our Introduction extensively to clarify our goal for the paper. As a result, we make the case in the introduction that considering steady-state dynamics is appropriate for our goal, while recognizing other published work that addresses non-steady-state cases. Third, in the section entitled "Specifying $f(\tau)$", we have acknowledged conceptualizing the importance of a tailed water age distribution (rather than a power law *per se*) as critical for our discussion. We now state explicitly that the goal of conceptualizing and visualizing the relationship among exchange, storage, and water age can be accomplished using either a power-law or exponential function, because either adequately represents the tailed distribution. Finally, we have modified our discussion of the "time-scale dependence of measured/modeled rates of hyporheic exchange" in a way that steps away from the power-law assumption and associated estimates of τ_0 and τ_n. Instead, we emphasized that our discussion point is not predicated upon the existence of a power-law, but rather is illustrated by our consideration of a power-law. We have chosen to avoid delving into discussing the literature that investigates the limits of integration because we believe this is tangential to the intended message of our paper.

R2 Comment 1, part 2

While the purpose of this simulation study is largely expositional in its aims, I believe it would be more informative if the authors enhance their discussion on the mechanisms of water transport in subsurface aquifers. I share the authors' motivation of accurately estimating hyporheic exchange flows, subsurface RTDs, and aquifer storage in order to use these metrics within a broader framework that would improve estimates of stream bioreactivity or some other ecosystem property. For this reason I encourage the authors to help the reader understand the mechanisms that may control the parameters they use to determine physical transport. The authors devote a good amount of text to explain the relation between the RTD and the magnitude of hyporheic exchange fluxes (with fixed storage and integration limits), but the range of power law exponents explored would encompass a range of different transport mechanisms that ultimately result in completely different physical processes governing hyporheic transport. An α of 1.9 borders on a "thin-tailed" distribution that could

be approximated with an exponential RTD, which represents a well mixed system (sensu Bencala and Walters, 1983). For an aquifer with constant storage and subsurface properties on the scale of Figure 2, this would represent very rapid transport and (likely) turbulent mixing in a coarse grained aquifer. It is unrealistic to assume that the bounds of integration on the RTD would be the same as in a system described by an RTD with α *= 1.3*

We agree that a discussion of the mechanisms that control values of α represents an important contribution to the literature. However, we question whether such a discussion furthers the goal of this paper. After considering this question, we have concluded that an in-depth discussion of mechanism associated with varying values of α drives away from the main thrust of the paper (considering the ecological importance of a tailed water age distribution). Further, we conclude that such a discussion may be premature – at least given our understanding of the state of the science.

Specifically, we are not sure that we agree with the reviewer's interpretations of α. For instance, while an α of 1.9 would indeed represent a well-mixed (i.e., relatively homogeneous) system, we don't think that a system on the scale of that shown in Figure 2 could have an α of 1.9 – precisely because such systems are not well mixed due to their scale. As evidence, we point to Table 1 in our manuscript and notice that the lowest values of α are associated with mountainous streams with morphology conducive to hyporheic exchange – where hyporheic zones are likely to be most expansive.

Given our concern that a more in-depth discussion may be off point, and the fact that we and the reviewer have differing opinions of how we might interpret α, we point to paragraph 6 of our discussion as a place where we recommend research investigating stream characteristics that influence values of α as a productive line of future inquiry. Here, we also ascribe what meaning we are comfortable ascribing to the value of α, and point out that some knowledge of s may be important to inferring geomorphic or hydrologic meaning to values of α.

In the paper as a whole, the important point is that α varies among streams and controls the shape of the AD_s under the assumption of a power-law. That assumption aside, the shape of AD_s and the size of the hyporheic zone are, combined, the critical determinant of the magnitude of exchange in streams. We believe this message is clear in the 6th paragraph of our discussion and the message is reinforced in the conclusions.

While, again, we agree that the recommended discussion would be useful, we are not convinced this paper is the place do provide it, nor are we convinced that there is general agreement among hydrologists on how we might infer hydrologic mechanism from values of α.

R2 Comment 1, part 3

Mechanistically, it is known that both limits of integration of the power-law RTD are likely bounded by physical processes. For example, τ_0 *may tend to zero if the process is molecular diffusion molecular diffusion or transition to an exponential-like RTD due to rapid turbulent mixing in coarse grained streambeds (Roche et al., 2019); these are two example of cases where* τ_0 *is not explicitly "bounded by the scale at which geomorphic features can be represented by the model." Similarly,* τ_n *is set by some structural property of the aquifer such as the depth to bedrock or the advective timescale between geomorphic units (e.g., Herzog et al., 2019), which also structures the transition from power law to exponential decay of the RTD at late times.*

Given the (now more clearly articulated) goal of our paper, we have elected not to address the literature covering the limits of integration of the power law. We feel this delves into theoretical

hydrology, and pulls the message of the paper away form our intended scope. However, we have clarified in paragraph 9 of our discussion that the statement "bounded by the scale at which geomorphic features can be represented by the model" applies only to three-dimensional finite-element or finite-difference floodplain models, which make no assumption about the shape of a residence time distribution, but rather, model an emergent residence time distribution based by simulating 3D flow through geomorphic features.

R2 Comment 2

Unclear connection to coarse grained alluvial aquifers The reactor theory introduced here is quite general compared to more recent theory that has been developed for fluvial fate and transport. As such, it is unclear why the authors specify coarse grained aquifers throughout the study. I would expect that transport in such systems to be more dispersive than in finer grained systems for which the concept of nested flowpaths was developed, so it would be useful for the authors to provide additional details to clarify the connection to coarse grained aquifers.

As stated in our response to Reviewer 2, comment 1, part 1, we have removed references to expansive course-grained alluvial aquifers (ECGAAs) throughout the text.

R2 Comment 3

Aquifer storage is quite often unknown and very difficult to measure. Throughout the m.s., it is assumed that s is generally known and needed to find $q \downarrow$. *While I rarely work at the spatial scales discussed in this study, I have found very few (if any) studies where s is faithfully measured. Usually, the exchange flux is more easily quantified, and s is unknown (e.g., Harman et al., 2016), or both are unknown. The authors should include additional justification for why s is assumed known throughout this study.*

We agree that aquifer storage is quite often unknown. Yet the fact that exchange will increase in proportion to storage (if α is held constant) is a foundational concept that we think is important to highlight given the goals of our paper (see Equation 10 in the paper). We don't understand the comment suggesting that we assume s is known throughout our paper. In the section entitled "Visualizing hydraulic geometry," we describe a method by which s can be estimated under limited circumstance and then acknowledge it is largely unknown in other cases. To circumvent this problem and still provide visualization of hyporheic exchange, we then focus on a representative unit (RU) of the aquifer (where $s = 1$) for the rest of our analysis. In our discussion and conclusions, we point out that research designed to provide landscape-scale assessments of variation in s and α (or more broadly, s and the shape of the AD_s) would provide new understanding of the role of hyporheic exchange in stream networks. Finally, in the paper, we question whether quantification of exchange flux is consistent across methods, given that different methods are associated with different time scales of water age.

Based on a later comment (see below), we have reworked our definition of the representative unit (RU), and believe this is the best way to clarify our paper so that a misreading of the paper similar to that which yielded this comment is less likely occur.

R2 Comment 4

Improve connections to existing literature. I applaud the authors' encouragement of greater integration of engineering principles into ecological theory, but they would serve the reader if they

acknowledge the wealth of existing work that has already used principles from reactor theory to improve models of hyporheic exchange, reactive transport, and ecological theory. Specific to hyporheic exchange, a good amount of work aiming to identify mechanistic controls on RTDs is summarized in Boano et al. (2014) – including links to what the authors refer to as hyporheic geometry, to reactive transport, and to ecological theory. Connection between residence time distribution and mass removal (i.e., nutrient spiraling) is, to my knowledge, first addressed in a model by Runkel (2007) using the phenomenological transient storage model, though this idea has been pushed forward by many others (e.g., see works by Tonina, Cardenas, and Bolster). The authors may also be interested in the developments of rSAS theory (Harman, 2015) and its application to hyporheic zone storage (e.g., Harman et al., 2016), since rSAS theory represents a generalization of the mathematical concepts introduced here.

We thank the reviewer for pointing out our oversight of rSAS. Given the goal of our paper, we assumed that the need to start with steady-state assumptions would be self evident, and therefore did not address rSAS and the necessary complexity it invokes. In hindsight, we can see this was a mistake to avoid citing rSAS entirely. We have extensively modified our introduction to clarify the goal of the paper. In the introduction, we now address rSAS directly, and refer the reader to appropriate citations to the rSAS framework to find a similar mathematical treatment that addresses the non-steady-state case.

We have now included a cite to Boano et al., although we note there are several such reviews of the importance of hyporheic exchange. We had cited Boulton's 2010 treatise, which covers a lot of the same material. Boano's paper, though, does cover the mechanisms of hyporheic exchange more thoroughly, and we have happily added it to our citation list.

We are confused by the reviewers suggestion that we cite Tonina, Cardenas, and Runkel, as we have cited each of those authors – although clearly only selected papers produced by each. We are also confused by the suggestion to cite nutrient spiraling work. The lead author of our paper (Poole) has published nutrient spiralling models for more than 15 years and is aware of no current link between hyporheic geometry or reactor theory and nutrient spiralling theory. We now have 46 citations in the paper, approximately 1/3 of which are from the ecological literature and 2/3 from the hydrologic literature.

R2 Other Comments

- *C05 (Abstract): The main text does not describe how the "...nuanced and realistic understanding" provided in this study differs (or improves) upon current paradigms in the fields of aquatic research or resource management. Perhaps a comparison of existing approaches would help these communities better understand how they can use this theory to improve predictions, e.g., through better designed field experiments.*

  Our paper includes a section entitled "An illustrative application: water temperature." In that section, we demonstrate how the theory can be used to think about hyporheic influence on water temperature. We revisit this in paragraph 7 of the discussion, and reference the example in the conclusions. We are unsure how this example doesn't demonstrate how the "nuanced and realistic understanding" of hyporheic hydrology can improve conceptualization of otherwise fuzzy concepts (e.g., as presented in reviews of hyporheic influences on stream ecosystems).

- *C06 (L23): Hyporheic discharge is more commonly referred to as hyporheic exchange flux.*

We disagree. The word flux implies the integral of all of the exchanges across a surface. The phrase "hyporheic exchange flux," we believe, implies a net exchange. Under the heading "Conceptual Model," in the third paragraph, we have defined our terms. We say that we adopt the reference frame of hydrogeologists, in which case the flux loss from the aquifer to the stream is "hyporheic discharge" and the flux gain by the aquifer from the stream is "hyporheic recharge". The sum of these two components would be "hyporheic exchange flux," by our reading of the term.

- *C07 (L106): Terms such as isometric and unipartite will would stand to be translated if the authors are aiming to maximize adoption by aquatic researchers and water managers.*

  We have adopted friendlier language.

- *C08 (Figure 2): It is unclear how this figure was generated. What is meant by the distribution of surface water? Are advective flows considered to be moving at constant velocity, or is a physical model being used? What hyporheic zone properties are being used?*

  We have improved the figure legend to say that the purpose of the figure is simply to visualize the non-contiguous nature of TSZs in an aquifer. The figure legend now states that the visualization is derived from an application of the HydroGeoSphere model on a restoration site on Meacham Creek in Oregon, USA.

- *C09 (L212-215): This explanation is undercut by the fact that the choice of limits does influence your model results substantially, as mentioned in the Discussion. Consider replacing "for other purposes" with a brief acknowledgment of what implications the choice of integral limits has on model outcomes, since this is the intuition you hope the reader will gain.*

  We have modified this sentence to say that the values are inclusive of residence times typical of the systems we study and included a citation.

- *C10 (L216): $f(\tau)$ is confusing because this notation is already used to denote the shape of the function used to create the pdf. Consider using a different variable.*

  We are unsure how to make this text clearer. Up until this point in the paper, $f(\tau)$ references the shape of an undefined function. This is intentional, because the equations up to this point will work with any definition of $f(\tau)$. Expressions 11 and 12 tell the reader how $f(\tau)$ is defined for the remainder of the paper. So the notation is not confusing but rather it is precise. We are, in fact, defining $f(\tau)$, which has up to this point in the paper, been intentionally undefined. When we first introduce $f(\tau)$ under "Quantifying Hyporheic Hydraulic Geometry", paragraph 3, where we state "If we choose a function, $f(\tau)$ to represent the desired shape of AD_s..." which tells the reader explicitly the purpose of the notation $f(\tau)$.

- *C11 (L274): Note that the empirical or mechanistic relationships mentioned here must be linear for this theory to hold, which implies first-order reactions. That may be a bit of a stretch for microbial community composition, though I am unfamiliar with microbial ecology theory.*

We can understand how some readers may jump to this conclusion because the assumption of first order reactions is so commonly a limitation of approaches that use integration. However, the temperature example provided in our paper explicitly demonstrates a case where a non-linear relationship between τ and some aspect of hyporheic water (i.e. temperature, Fig 6) is used with our approach. We have clarified in the text that any relationship, linear or non-linear, can be used. We have also moved text that was formerly included where Eqs 13 and 14 are introduced into the the discussion of Eqs 13 and 14, so that the discussion of these equations is consolidated into one location.

- *C12 (L329): "...determining the magnitude of $q\downarrow$ within this model framework."*

  Here the reviewer seems to be suggesting that s influences the magnitude of hyporheic exchange only within our modeling framework. We have opted not to incorporate the suggested change. Our point is that s must influence the rate of hyporheic exchange. Even in real-world systems, larger hyporheic volumes will be associated with greater exchange. The reviewer's point may be that residence time distribution could also adjust to a change in hyporheic volume and, in real world systems, the relationship between exchange rate and s may not be linear (as depicted in Eq 10 assuming α and therefore $W(\tau)$ does not vary). However, at this point in the discussion, we don't claim the relationship is linear. We simply make the point that exchange rate is related to s – in both our modeled system and in the real world.

3 Main Comments from Reviewer 3

R3 Comment 1

NOVELTY SPECIFICATION: As I said, the manuscript does not present novel concepts (and this is correctly recognized in the text), so it is fundamental to state the key contribution of the work. In my view, the contribution is to choose a specific type of RTD (i.e., a one-parameter power law distribution) which is considered to be representative of hyporheic exchange, use this RTD to develop expressions for relevant quantities such as fluxes and age distributions (AD) as function of a few parameters (alpha and s), and suggest to employ this framework to classify streams by linking alpha and s to the characteristics of streams and catchments. If this was the intended aim of the manuscript, I recommend it to state more openly because it now gradually emerges from the text, and it is not evident to which extent the use of power law RTD is a mere example or a relevant assumption.

This was an especially helpful comment. We have substantially restructured both the abstract and the introduction to clarify our intent, and believe the remainder of the paper is now framed more precisely. We are grateful to the reviewer for this observation.

R3 Comment 2

APPLICATION EXAMPLES: It is stated (line 359) that "chemical engineers have been applying these equations to bio-reactors (such as sewage treatment plants) to predict whole-system operation for decades [...]. Thus, a view of "streams as bioreactors" may provide a launchpad for potential collaborations between the engineering and ecological disciplines". It would then be extremely valuable to provide example of the applications. However, the applications of the theory that are suggested are not always very informative (e.g., temperature dynamics in fig 6; age of

upwelling water in fig 4 works better), and I think that the reader is left out questioning how useful the proposed framework is. If a new approach is proposed, compelling examples should be provided. The same is true for Fig 7: most of the comments are about the limitations stemming from the assumptions, which is fine, but I would also stress the take-home message from the example.

The reviewer is correct that the temperature application is intended to provide a simple, accessible illustration of the utility of considering tailed residence time distributions and "hyporheic hydraulic geometry" for scaling field observations to ecosystems. We have clarified the text introducing Eqs 13 and 14, and consolidated the discussion of the same in the "Discussion" section. Upon reflection, we have also removed the text quoted by the reviewer, above. The text quoted by the reviewer was perhaps over-inflated and distracted from the main message of the paper.

R3 Comment 3

CLARIFICATIONS: in a number of points, the text could be more straightforward and point out the aims of the concepts that are introduced, which are not always clear enough. Some examples follow:

- *I think that the role of short residence times is overemphasized when it is said that "if we are willing to accept that hyporheic exchange scales according to a power law [...] our application of the equations assuming a power-law RTD reveals [that] the rate of hyporheic exchange in any given stream is dependent on the range of water ages considered" (lines 364-367). Starting from this point, almost a half of the discussion section deals with the implications of the power-law RTD; however, it is particularly the tails of RTDs that have been found to have power-law behavior, while this is not necessarily true for small times. In particular, the fractal behaviour of morphology (which leads to the fractal, nested system of hyporheic flow cells; line 387) should break up at small spatial scale due to physical constraints (e.g., at grain scale for topograpy-driven exchange, or at the Kolmogorov scale for turbulence-driven exchange). Moreover, at small scale different physical processes may prevail (i.e., diffusion rather than advection). What I am implying is that the role attributed to small residence times is likely to stop below some threshold time scale when the RTD may no longer be described by a power law. Because the manuscript is directed also to readers that are not familiar with hyporheic RTD, I think that the message "RTDs are always power-law distributed" could be misleading and the concepts expressed here may derive from the abnormal behaviour of the power-law distributions at small times when they could no longer be a good approximation of actual RT.*

  We agree that this section over-emphasized the power-law. Inappropriately, we had used "power-law" as a synonym for "tailed distribution," which contributed to the consternation reflected in this review comment, and to similar discomfort from the Reviewer 2. We have reworked this text to emphasize our assertion of time-scale dependence of exchange rates, while decoupling the message from the assumption of a power-law.

- *The representative unit (RU) is defined at line 185 as "a conceptual unit of hyporheic water storage (s=1) which has, by definition, the same RTD and AD as the larger hyporheic zone it represents." This is probably not the best definition as it may lead to think that RUs are physical parts of the hyporheic zone (HZ), while it is unlikely that a portion of the hyporheic zone has the same RTD and AD than the total hyporheic zone. Later on it becomes clearer that the RU is introduced only to discuss the properties of HZ regardless of volume, i.e., an idealized HZ with unit volume. While the definition is formally correct, it does not make clear*

why the concept is introduced, leading to potential confusion for the reader.

This is an excellent point. We have adopted the suggested definition of the RU and believe this improves the clarity of the paper.

- *the manuscript "suggests that research efforts to identify stream characteristics correlated with values of alpha and s would expand opportunities for rapid characterization of hyporheic hydrology across stream networks [...], especially if such correlates were obtainable from readily available spatial data sets (digital elevation models, LIDAR, aerial photography, etc.)" (line 331-335). It is worth stressing the temporal variations of streamflow or groundwater flow also affect hyporheic exchange and should be factored when these data sets are built, or otherwise we would improperly attribute observed variations in hyporheic exchange to spatial rather than to temporal drivers. The picture is of course complex, but I think that it is important not to oversimplify it in this point.*

  This is an important point and we have added a sentence and citation acknowledging the role of temporal variation in stream discharge.

- *The notation is changed at line 221 by renaming many variables. Is this necessary? If this notation is simpler than the former one, why not using it throughout the manuscript?*

  We have clarified in the text that our intent was not to redefine the notation, but instead, to define initially the notation used to describe characteristics of TSZs.

- *It is stated at line 197 that "For the remainder of this paper, we assume that the hyporheic RTD is proportional to a power law with a negative exponent" – how critical is this assumption for the manuscript? See main comment 1.*

  We have changed the text to reflect that the assumption of a power-law is not necessary, except insofar as we need to assume a shape for $f(\tau)$ in order to create our visualizations (e.g., Figs 4 and 5)

R3 Other Comments, by Line

- 7 *"expansive coarse-grained alluvial aquifers (ECGAAs)" – I do not know what an expansive aquifer is. I recommend to explain it, even briefly.*

  We have removed references to ECGAAs.

- 73 *it is said that q can represent, among other things, the "rate at which the cross-sectional area of hyporheic water exchanges with the cross-sectional area of the channel". This description is confusing (water flows through the cross section, and it is not straightforward where it is exchanged between channel and hyporheic zone). I suggest to describe it as the rate of water exchange per unit river length, which I think is a correct description.*

  This is a fascinating comment. Many models of hyporheic exchange use the concept of a_s/a, or the ratio of the cross-sectional area of hyporheic water to the cross sectional area of the stream. Exchange is in dimensions of $[\mathrm{L}^2][\mathrm{t}^{-1}]$. Such models assume that the 2D

representation of a stream system is a cross section. The reviewer is thinking about a 2D longitudinal section – something we have never considered.This is clearly a legitimate way of thinking about hyporheic exchange. However, this model would not consider the rate of exchange per unit river length (which would be be 1D exchange measured in dimensions of [L][t^{-1})]. Instead, such a conceptual model would consider the 2D rate ([L^2][t^{-1}]) of water exchange between a longitudinal section of channel area (steam depth times reach length) and a longitudinal section of the hyporheic zone (thickness times reach length times porosity). We have re-framed to the 2D case to refer to "cross- or longitudinal-section area" of channel and hyporheic water.

- 122 *"exit age density function" – I think it is more coherent to refer it as residence time (RT) rather than age, since it has been already stated that RT denotes the time when water leaves the aquifer.*

  "Exit age density function" is the definition provided by Cocker 2001 in the cited chemical engineer text, and corresponds to the notation $E(\tau)$. Due to confusion in the term "residence time" in the published literature (described in the new section of the paper "Water age distributions"), we have eliminated the term "residence time" from the paper.

- *144 it would be useful to specify that W(t) is dimensionless, as the dimensions of other quantities have been reported.*

  Added "[dimensionless]" in sentence describing $W(\tau)$.

- *153 missing "n" in "functioN"*

  Corrected.

- 177 *this section is titled "Visualizing hydraulic geometry" and the next one "Visualizing hyporheic hydraulic geometry". However, they both refer to the aquifer exchanging water with the channel, so the difference between the subject of the section is unclear. If there is no difference, titles should be changed.*

  We have consolidated this under a single heading.

- 181 *"s can be approximated as the product of aquifer dimensions (length, width, and depth) and aquifer porosity." – This is true only if s represent a volume, but as said before it can have different definitions. I suggest to better specify it here.*

  We have modified this text to reflect the possibility of different dimensions.

- 209 *remove parentheses before and after "tau $<$ 60 s".*

  Corrected.

- *Fig.3 I am not sure how informative this figure is.*

  Although the figure is not informative regarding the overall shape of the power law, our intent is to help the reader visualize how τ_0 and τ_n influence the area under the curve.

- 240 *"hyporehic" should be "hyporheic". Same at line 340.*

  Misspellings have been corrected.

- 250 *"residence time" should be "water age"*

  Corrected.

- 311 *"I(t) is not the same as, but can be derived from E(t)" – is the other way around also true, as stated at line 445?*

  To address other comments, the referenced text has been removed from the revised manuscript.

- 337 *missing space after "hereafter"*

  Corrected.

- 340 *"if more complex channels have higher rates of hyporehic exchange, such channels are likely to be associated with larger values of alpha." – This is true only if the volume s is the same, while comparing river systems of different sizes could lead to very different results. I would rephrase slightly to avoid ambiguities.*

  Addressed.

- 355 *"Considering multiple transient storage zones within the context of hyporheic hydraulic geometry provides the potential for a quantitatively rigorous but practical mechanism for scaling hyporheic heterogeneity to whole stream networks." – the use of multiple transient storage zones is essentially a technical way to discretize water ages in finite classes. It is not very different than, e.g., using finite time steps in a particle tracking approach, or a discrete cells in a finite difference methods, and I would present it as such. Moreover, the link on how it can allow for up-scaling is not well defined (see also main comment 2).*

  The reviewer make an excellent point. Our intended meaning was that consideration of "the age distributions of hyporheic discharge and storage..." is useful for scaling. Multiple TSZs is just one way to consider those distributions. We have adopted the more general language. Also, this text now exists in a consolidated discussion of the temperature example, which helps underscore that the temperature example illustrates the utility for up-scaling.

4 Responses to Other Reviewers

Reviewer 1

This manuscript analyzes travel-time distributions in the hyporheic zone under the assumptions of steady state and negligible net gain or loss of groundwater. The authors emphasize that the age distribution of the water returning to the stream differs from the age distribution within the hyporheic zone. However, this has already been known. I had really hard times figuring out what's new in the manuscript.

We agree that the contribution of the manuscript is not in describing that these distributions are different, but rather, in: 1) the presentation of the mathematical model – as applied to hyporheic zones and in a way that is more accessible than other treatments of the same material; 2) the visualization of how the mathematics can inform a more accurate conceptual understanding of hyporheic exchange; and 3) describing how the consideration of water age distributions can be useful for scaling field observations to ecosystems. We believe our revised introduction now highlights the contribution of the paper.

R1 Comment 1, part 1

The authors motivate their study by the analysis of expansive coarse-grained alluvial aquifers (for which they introduce the completely unnecessary acronym ECGAA). I doubt that the hydrology of these large gravel bodies is ever at steady state, and I also doubt that neglecting any net groundwater upwelling or permanent loss of streamwater to groundwater is appropriate.

We have removed references to ECGAA's. Based on 20 years of experience working in such systems, the evidence suggests these aquifer levels respond to changes in river discharge within hours and are roughly in steady-state any time river flow is stable.

R1 Comment 1, part 2

However, under the given restrictions, the relationship between travel times (also denoted transit times) and groundwater age can be looked up in Liao & Cirpka (2011, doi: 10.1029/2010WR009927, Appendix A), which is most likely not the first such derivation. Note that a formulation of hyporheic exchange that are based on transit/travel-times have already been proposed by Wörman et al. (2002, doi:10.1029/2001WR000769) if not much earlier, whereas formulations using the age distribution/memory function are at the heart of multi- (or single-)rate mass transfer formulations.

We agree with these assertions. Our intent is to make the implications of these relationships more accessible to those who consider hyporheic zones conceptually, rather than mathematically. Again, the changes to the introduction should help clarify this point.

R1 Comment 1, part 2

Under the given conditions, you get one from the other by taking derivatives or integration. To complete the description of exchange, you either need the volume of the hyporheic zone (relative to the volume of the stream), or the exchange-rate coefficient. Period.

We agree. In the first sentences of the section of the manuscript entitled "Visualizing hydraulic geometry," we acknowledge that an estimate of s is necessary to determine $q_{\downarrow}$, yet often sometimes difficult to obtain. We then analyze an aquifer of a unit volume (representative unit; $s = 1$) to build our visualizations.

R1 Comment 2

The notation preferred by the authors is confusing. The term residence time distribution is used by other authors as the age distribution. Please read the landmark paper of Botter et al. (2011, doi:10.1029/2011GL047666). This paper also discusses the impacts of transient flow (for catchments rather than the hyporheic zone, but their master equation could of course be adapted). It

would be interesting to see how the concept of StorAgeSelection functions (Rinaldo et al., 2015, doi: 10.1002/2015WR017273) could be used to describe systems with extensive hyporheic exchange. But obviously, the authors are not aware of these concepts.

We believe that Harmon and colleagues (2016) have applied the rSAS framework to hyporheic zones in their paper entitled "How does reach-scale stream-hyporheic transport vary with discharge? Insights from rSAS analysis of sequential tracer injections in a headwater mountain stream" and would point the reviewer to that paper to see how the concept can be applied to hyporheic systems. We had chosen not to invoke rSAS in our paper, because the goal and scope of the paper was intended to provide the simplest mathematical model possible in order to provide the reader insight into how water age distributions work and why they matter. Our goal was to help non-hydrologist aquatic scientists integrate more hydrologic realism into conceptual models of hyporheic exchange. We acknowledge that our paper makes only a limited contribution to the pure hydrologic literature, which is why are are submitting to a more general journal rather than, e.g., "Water Resources Research."

R1 Comment 3

Another odd term used by the authors are "isotemporal surfaces". These guys are known as isochrones, a very established concept in hydrology.

We agree that the term isochrones is commonly used to describe a line on a two-dimensional representation of, e.g., water age. Our representation is three-dimensional, and therefore surfaces (rather than lines) are isochronal. We have settled on the compromise "isochronal surface."

R1 Comment 4

As application, the authors chose the temperature regime of the extended gravel plains. Too bad, heat is the one extensive state variable in shallow groundwater for which I would definitely NOT rely on advective travel times. Conduction into the transverse direction leads to a comparably strong thermal exchange among streamtubes, causing solute travel times to be different from "temperature travel times", and even worse making heat exchange with the land surface a relevant process. The conceptual model of the authors is that the hyporheic zone is thermally isolated from the land surface, that transverse exchange can be neglected, and that the classical Stallman (JGR 1965) solution on the one-dimensional propagation of sinusoidal temperature variations holds. You'd better start with the 3-D heat balance equation with realistic boundary conditions. It is very likely that the diurnal signal of temperature cannot be explained by the travel times of solutes plus the Stallman solution.

We have been studying and modeling heat transport in expansive coarse-grained alluvial aquifers for nearly two decades. We have published empirical observations from these systems that show strong relationships between residence time and aquifer temperature (Helton et al. 2012). While we agree that transverse exchange can not be neglected in mechanistic models, we base our temperature example on empirical relationships, where the effects of transverse exchange are baked in. Granted, we agree that the Stallman (1965) solution does not hold, but that doesn't mean that water temperature isn't related to water age. Although we cite studies that show empirical relationships between temperature and water age, the reviewer seems unaware these relationships in large alluvial aquifers. For many research applications, we apply and applaud the 3-D heat balance equation

championed by this reviewer. However, 3-D mechanistic approaches simply can not be scaled to river networks. In contrast, our simplified approach, based on field observations of relationships among residence time and aquifer properties, is scalable. We disagree with the reviewer's assertion that simpler modeling approaches have no place in the scientific endeavor.

R1 Comment 5

Let me finish with my bewilderment on the statement "that integrals and probability density functions are typically presented more thoroughly in engineering curricula than in ecological curricula" (lines 42-43). What type of an argument is that? You can expect basic calculus and probability theory from any quantitative scientist (and I would add linear algebra, vector calculus, and differential equations to the mix). Otherwise it's not science. However, if the authors want to address readers without any math background, their equations won't help either.

We disagree with the premise that work not anchored in calculus, probability theory, linear algebra, vector calculus, and differential equations is "not science." Rather, we believe that those without extensive mathematical training can grasp the equations we present, assuming the equations are presented in a manner that doesn't presume the mathematical aptitude of this reviewer. We point this reviewer toward the comments of Reviewer 4 as an example of a scientist who benefited from our treatment of the material.

Reviewer 4

In this work the authors present a quantitative description of hydraulic geometry to visualize the interdependence among hydrologic variables such as age distribution and residence time distribution in the hyporheic zone. This is important because concepts related to AD and RTD are often misunderstood and misinterpreted and this paper will help to clarify and distinguish between these two variables. My own research pertains to hyporheic hydrology and I myself struggle with this distinction.

Overall, I very much enjoyed this paper and the rich development of equations describing the relationships between hyporheic flow, residence time, and storage. This paper is well-written, the theoretical constructs are well-developed and described, and the theme of the paper considers aspects of hyporheic zones that relate to scaling constructs that will be useful for non-modeling readers.

A main theme within this paper is to clarify differences and elucidate interdependencies between AD and the RTD. Lines 308-312 is one example where this distinction is highlighted and it has been noted elsewhere throughout the paper. Despite this prominent theme, after reading the paper, I still did not have a better understanding of how to conceptualize and understand the differences between the two. The authors are entrenched in the theoretical constructs and spend the majoring of the paper on this. Because of this, the conceptual development, and clear articulation of what these metrics actually mean in real life is lost. The temperature example did not help to resolve this issue for me. My suggestion is to resolve this using Figure 1. In Figure 1, you have already done the work showing TSZs in series, so can you provide a simple example of min/max ages for each TSZ within your conceptual model and associated residence times? And show how the pdf of the AD/RTD changes as a water particle moves through the TSZ. A simple example within your conceptual model would help distinguish between the two for the ecological/hydrological audience.

In order to clarify how hyporheic discharge and storage can have two different water age distributions, we have inserted a short section at the beginning of the methods section entitled "water

age distributions" to anchor the reader in this concept prior to introducing our conceptual model. Although we could attempt to integrate the distributions in cartoon form in Figure 1, as suggested by this reviewer, we have provided Figure 5, which incorporates the relationship between water age, discharge, and storage as a visualization of actual model results rather than a conceptual cartoon.
