# Supplementary material for: Hyporheic hydraulic geometry: Conceptualizing relationships among hyporheic exchange, storage, and water age
Source: PLoS One. 2022 Jan 14;17(1):e0262080. doi: 10.1371/journal.pone.0262080 (PMC8759689; doi:10.1371/journal.pone.0262080)
Supplement: Attachment — Submitted filename: Second_Revision_Response_To_Reviewers.pdf [file pone.0262080.s004.pdf]

Response to Review Comments

Geoffrey C. Poole, S. Kathleen Fogg, Scott J. O'Daniel, Byron E. Amerson, Ann Marie Reinhold, Samuel P. Carlson, Elizabeth J. Mohr, and Hayley C. Oakland

December 15, 2021

1 Associate Editor Comment

After careful consideration, we feel that it has merit but does not fully meet PLOS ONE's publication criteria as it currently stands. Therefore, we invite you to submit a revised version of the manuscript that addresses the points raised during the review process.

More specifically, I encourage you to take into consideration the concern of reviewer 3 on their second issue: lack of application examples. I wonder if another example could be provided to tackle this issue and make the manuscript less speculative.

We included an application example using a conservative tracer data, as outlined below in our response to the main comments of Reviewer 3.

Additionally, although not in response to review comment, we made a small adjustment to the title in an effort to more precisely articulate our goal for the paper. The adjustment is shown in the red-line/strikeout version of the manuscript.

2 Reviewer 3: Main Comments

In my previous review I identified three issues that were in need of attention: (1) insufficient novely specifications, (2) lack of application examples, and (3) need to clarify some paragraphs. The revisions made by the authors have substantially improved the manuscript in terms of issues (1) and (3): the introduction is now better structured and helps the reader in understanding the aim of the concepts later described in the manuscript, and many passages are now clearer. In terms of issue (2), I still think that a manuscript that aims to provide a framework to help interpreting natural processes would greatly benefit from an example of application of this framework that is not purely theoretical. At present, the provided examples (fig. 4-7) are obtained from model simulations, and the illustrative example of water temperature is no different. I am not sure if the case study from which fig.2 was derived could provide such an applicative example, but in that case (or with any field data) the potential impact of the manuscript would greatly increase. Otherwise the manuscript is much more speculative, and I leave to the Editor the choice if the manuscript can be considered without this application.

To incorporate an empirical element, we conducted a tracer experiment in an annular flume and assessed the response of specific conductance to the addition of a salt slug. We describe our data collection effort in the new section of the paper entitled "A visualization from conservative tracer data." We have also added a complementary paragraph to the discussion near line 427 in the manuscript, outlining the take-away points from our analysis of the experimental data.

Reviewer 3: Other Comments

- *6 "the top few hundredths of a meter" - I would say "top centimeters", but this is a matter of taste.*

  When describing scaling across orders of magnitude, we prefer to maintain constant units. Therefore, we have maintained our original wording.

- *46 "rSAS" is firstly introduced here, and the acronym should hence be defined.*

  Added "ranked StorAge Selection" to sentence before "rSAS"

- *95 "a hydrosystems" - "a hydrosystem".*

  See line 82, we removed plural.

- *Fig.6: the last sentence of the caption is missing something ("greater water ages and ."). Remove "and" or complete.*

  We removed "and".

- *493 "While this problem may seem somewhat intractable" - I think that my previous comment to this part was not completely clear: I understand the point the authors are raising, i.e., the method of analysis may lead to underestimating the flux because of the chosen scale. What I meant is that a low scale probably exist in most situation, because the RTD is unlikely to assume infinite values for small values of residence times, as the discussion here seems to imply. This is true for different types of RTD (e.g., for both power law and exponential distributions): at small residence time, the increase in probability may break up. I simply suggest to avoid giving the impression that the "true" flux can never be found even if a very fine scale of analysis is employed.*

  The essence of the reviewer's comment seems to be encapsulated their final sentence. The reviewer seems to be uncomfortable with the idea that the scale-dependence of hyporheic exchange could continue to exist at, say, even the molecular scale. We don't understand the review's discomfort with this idea, though we acknowledge that the evidence necessary to refute or support the assertion does not exist. To address the reviewer's primary concern, we eliminated the word "knowable" from the following sentence: "... we offer caution against the assumption that there is a single, knowable, time-scale independent rate of hyporheic exchange within any stream channel." The remaining text emphasizes that observations of hyporheic exchange will differ when different water age timescales are included or excluded, a fact with which the reviewer appears to agree.

  We have also made other minor edits to this section to soften the language or make our argument more precise, as noted in the red-line/strikeout version of the manuscript. In the end, while we disagree with the reviewer, we have ensured of our text "avoids giving the impression that a 'true' hyporheic flux can never be found," as requested.
