# Supplementary material for: Correction: Hyporheic hydraulic geometry: Conceptualizing relationships among hyporheic exchange, storage, and water age
Source: PLoS One. 2024 Aug 12;19(8):e0308936. doi: 10.1371/journal.pone.0308936 (PMC11318846; doi:10.1371/journal.pone.0308936)
Supplement: S1 Appendix — (PDF) [file pone.0308936.s001.pdf]

Supporting information

S1 Appendix. Assuming the water age distribution follows a power law with a minimum water age τ_0 and a maximum water age τ_n (using Eq 12 from the main text as the definition of $f(\tau)$), the solution for $E(\tau)$ is:

$$E(\tau) = \frac{(\tau^{-\alpha} - \tau_n{}^{-\alpha})(-\alpha+1)}{\tau_n{}^{-\alpha+1} - \tau_0{}^{-\alpha+1} - \tau_n^{-\alpha}(\tau_n - \tau_0)(-\alpha+1)}. \quad (1)$$

The solution for $W(\tau)$ is:

$$W(\tau) = \frac{(\tau_n{}^{-\alpha+1} - \tau^{-\alpha+1}) - \tau_n{}^{-\alpha}(\tau_n - \tau)(-\alpha+1)}{(\tau_n{}^{-\alpha+1} - \tau_0{}^{-\alpha+1}) - \tau_n{}^{-\alpha}(\tau_n - \tau_0)(-\alpha+1)}. \quad (2)$$

The solution for the integral in Eq 9 (which can be used to solve Eq 7) is:

$$\int_{\tau_a}^{\tau_b} W(\tau)\, d\tau = \frac{\frac{\tau_n{}^{-\alpha}(-\alpha+1)(\tau_b{}^2 - \tau_a{}^2)}{2} - \frac{(\tau_b{}^{-\alpha+2} - \tau_a{}^{-\alpha+2})}{-\alpha+2} - \tau_n{}^{-\alpha+1}(\tau_b - \tau_a)(-\alpha)}{(\tau_n{}^{-\alpha+1} - \tau_0{}^{-\alpha+1}) - \tau_n{}^{-\alpha}(\tau_n - \tau_0)(-\alpha+1)}. \quad (3)$$

Assuming the water age distribution follows an exponential function:

$$f(\tau) = e^{-\sigma\tau} - e^{-\sigma\tau_n} \quad (4)$$

the solution for $E(\tau)$ is:

$$E(\tau) = \frac{\sigma(e^{-\sigma\tau} - e^{-\sigma\tau_n})}{e^{-\sigma\tau_n}\left(e^{-\sigma(\tau_0-\tau_n)} - \sigma(\tau_n - \tau_0) - 1\right)}. \quad (5)$$

The solution for $W(\tau)$ is:

$$W(\tau) = \frac{e^{-\sigma(\tau-\tau_n)} - \sigma(\tau_n - \tau) - 1}{e^{-\sigma(\tau_0-\tau_n)} - \sigma(\tau_n - \tau_0) - 1} \quad (6)$$

The solution for the integral in Eq 9 (which can be used to solve Eq 7) is:

$$\int_{\tau_{i-1}}^{\tau_i} W(\tau) = \frac{\sigma(\tau_a - \tau_b) + e^{-\sigma(\tau_{i-1}-\tau_n)} - e^{-\sigma(\tau_i-\tau_n)}}{e^{-\sigma(\tau_0-\tau_n)} - \sigma(\tau_n - \tau_0) - 1} \quad (7)$$
